# Chiral Separation via Molecular Sieving: A Computational Screening of Suitable Functionalizations for Nanoporous Graphene

**DOI:** 10.1002/cphc.201800413

**Published:** 2018-06-19

**Authors:** Samuel M. Fruehwirth, Ralf Meyer, Andreas W. Hauser

**Affiliations:** ^1^ Institute of Experimental Physics Graz University of Technology Petersgasse 16 A-8010 Graz Austria

**Keywords:** chiral separation, porous graphene, molecular sieving, nanoporous materials, racemic mixtures, force fields, tight-binding method

## Abstract

In a recent study [Angew. Chem. Int. Ed., 2014, 53, 9957–9960] a new concept of chiral separation has been suggested, which is based on functionalized, nanoporous sheets of graphene. In this follow‐up article we discuss the underlying principle in greater detail and make suggestions for suitable pore functionalizations with respect to a selection of chiral prototype molecules. Considering drug molecules as future targets for a chiral separation via membranes, the necessary pore sizes represent a big challenge for standard methods of computational chemistry. Therefore, we test two common force fields (GAFF, CGenFF) as well as a semiempirical tight‐binding approach recently developed by the Grimme group (GFN‐xTB) against the computationally much more expensive density functional theory. We identify the GFN‐xTB method as the most suitable approach for future simulations of functionalized pores for the given purpose, as it is able to produce reaction pathways in very good agreement with density functional theory, even in cases where force fields tend to an extreme overestimation of barrier heights.

## 1 Introduction

Mirror images of one and the same drug molecule affect biological systems in different ways. While one form may be beneficial for the health of the recipient, another could be extremely harmful. Therefore, it is an essential requirement of modern drug research to separate and test all enantiomers of bioactive substances.^1^,^2^ In most cases, a direct enantioselective synthesis is not feasible, and intermediate forms or final products need to be extracted from racemic mixtures. Contrary to common methods, a new concept of separation, based on chirally functionalized pore rims of essentially two‐dimensional membranes,^3^ reduces the whole process of separation to a single decision on the molecular level.

Currently, the enantioselective separation via chromatography is one of the most important techniques for the production of enantiopure compounds for the pharmaceutical, agricultural and food industries.^4^, ^5^, ^6^ Computational simulation has helped to gain insights into the process of molecular recognition.^6^, ^7^, ^8^, ^9^, ^10^ Several models have been suggested in the past in order to reduce the necessity of empirical rules and chemical intuition. According to the four‐point model,^11^,^12^ the chiral selector needs to interact with the reactant through four distinct contact points to achieve a chiral differentiation. In the special case of a surface‐mounted selector, which can only be reached from one side by the reactant, the number of necessary contact points reduces to three^13^,^14^ due to sterical hindrance. The applicability of the so‐called three‐point model^15^ has recently been tested by MD studies via simulated annealing.^16^ It was found that the enantioselectivity is constant for all homologues of the same mother compound obtained by changing one substituent of the chiral center, if a) this substituent by itself is not relevant for the three‐point interaction and b) does not affect the other three important interactions.

However, little is known about chiral resolution via two‐dimensional membranes. To the knowledge of the authors, this novel concept of enantiomer separation, originally suggested in Ref. [3], has not been proven experimentally yet. It is based on the fact that temporary bimolecular complexes, formed by an enantiomer of the target molecule and a carefully selected ‘gatekeeper’ molecule which is permanently attached to the rim of a pore, have a different spatial extension depending on the actual chirality of the target molecule. Therefore, the propagation of the enantiomer leading to a smaller temporary complex becomes more likely than the propagation of its chiral opposite. As a consequence, such a porous structure should be chirally selective. Note that differences in the binding energies of two enantiomers play a minor role for the separation in the proposed mechanism. The rate‐determining step is dominated by spatial hindrance, which is highly sensitive to the actual size of the temporary complex in comparison to the kinetic diameter of the pore.

We note that it needs to be clearly distinguished between membranes in the classical sense and membranes which are essentially two‐dimensional, i. e. membranes consisting of a single layer of atoms only. A typical transport process in the former is dominated by thousands of temporary adsorption scenarios, leading to either facilitated or retarded transport.^17^,^18^ The impact of the pore size on enatioselectivity in these structures has been studied recently for the case of three‐dimensional, homochiral metal‐organic frameworks (HMOFs).^19^ It was shown that high enantioselectivity is strongly correlated with a close match between the size of the pore and the size of the chiral sorbate. HMOFs are a novel class of microporous materials which are typically synthesized by the combination of metallic nodes with organic linkers in between.^20^,^21^ Up to now, successful synthesis has been reported only for a few systems.^22^, ^23^, ^24^, ^25^

However, a typical transport process in the latter case, i. e. through a single‐atom thick membrane, can be interpreted as a chemical reaction, a single molecular event of propagation through the pore, which either takes place or not.^3^ A potential material for this type of membrane are free‐standing sheets of graphene,^26^ which can be seen as the ultimate membranes due to their single‐atom thickness.^27^ Since a perfect sheet is impermeable to particles as small as helium, the necessary pores have to be created by various techniques.^28^, ^29^, ^30^, ^31^, ^32^ Common are either invasive post‐synthesis methods such as hole drilling with electron beams, or bottom‐up approaches such as the design of suitable precursors for the self‐assembling of the desired structures.^33^, ^34^, ^35^ Other post‐treatments are UV‐induced oxidative etching^36^, ^37^, ^38^ or ion bombardment.^39^,^40^

In the current study we formulate and answer two questions which we believe to be of largest importance for a future development of suitable, chirally active two‐dimensional membranes. First, we perform a series of calculations on a selection of organic, chiral molecules with typical functionalizations in order to determine which combinations of functional groups tend to give the largest size differences between ‘left‐left’ and ‘left‐right’ bimolecular complexes. In the former case, the target molecule and the gatekeeper molecule are of same chirality; in the latter case, they are of opposite chirality. This information is crucial for the proposed separation mechanism, as the complex size translates directly into a barrier height for the propagation of the target molecule. The pores of the two‐dimensional membrane are acting as molecular sieves for the temporally formed complexes. After swinging through the pore, the enantiomers are released on the other side of the membrane. Adsorption or desorption rates onto the gatekeeper molecule or anywhere else onto the membrane can be assumed to be in equilibrium on both sides since no covalent bonds are formed or broken during propagation from and to the pore. The actual transition, on the other hand, involves the crossing of a high energy barrier far from thermal equilibrium. Therefore, a pressure or concentration gradient has to be assumed in order to obtain a reasonable particle flux through the pore. Addressing this aspect of the separation principle necessitates a dynamical simulation which is planned for future studies. In the current work, we focus on a static analysis of the various complex sizes, interpreting this property as a descriptor for the expected barrier height in real propagation scenarios. An appropriate choice of the pore diameter, preferably in between the two sizes obtained for the two possible bimolecular combinations, will enforce a rate‐determining barrier which is almost entirely caused by spatial hindrance. This principle is in stark contrast to conventional separation processes, where retardation or reduced mobility are the consequence of different adsorption strength for different enantiomers.

The second question which we address in this article is of a more technical nature, aiming at the identification of the most suitable computational approach for a description of the proposed separation mechanism. From a computational chemistry point of view, this represents a big challenge due to the size of the systems to be simulated, paired with the need for highly accurate relative energies with respect to spatial hindrance upon molecular propagation. Even with structural relaxation left aside for a moment, a molecule propagating through any pore will show a more or less complicated reaction pathway, which is somehow related to the ‘corrugation’ of its electron density with respect to the reaction coordinate. In a simplified thought experiment, the latter can be thought of as the translation of the whole molecule in *z*‐direction, with the membrane being placed in the *xy* plane. In order to produce reliable predictions of selectivity and permeance, the method of choice should produce an energy profile which is as close as possible to reality. In a series of preliminary tests, we will use constrained DFT energy scans as reference profiles with a well defined reaction coordinate (i. e. the distance *z* from the pore center), and compare the force field and tight‐binding results against the DFT profiles, which are computationally more expensive by several orders of magnitude.

Our article is structured as follows. An overview of our computational approach is given in Section 2. In Section 3 we present our study on bimolecular complexes formed by organic, chiral molecules with typical functional groups and their suitability for a chiral separation based on size differences. Section 4 is dedicated to the investigation of approximative reaction pathways corresponding to the propagation of small organic molecules through nanoporous graphene materials, obtained with density functional theory, force field methods, and the GFN‐xTB approach. Having identified the most suitable method, we then revisit in Section 5 the system discussed in Ref.[3] where a chirally functionalized graphene pore is tested for the chiral resolution of 1‐aminoethanol. We extend the original study by small variations of the pore size in order to investigate the impact on selectivity and permeance.

## 2 Computational Methods

As a reference for energies and geometries of all systems studied in this article we employ density functional theory. The B97M‐V functional^41^ of the Head‐Gordon group is used as a reference for energies and structures throughout this article. This functional is a cost‐effective combination of local exchange and correlation following Ref.[42] with the nonlocal correlation functional of Vorhuis et al. (VV10).^43^ In the first study on complex formation we further apply B97‐D,^44^ a generalized gradient approximation functional including an empirical dispersion correction, as well as the well‐known hybrid functional B3LYP,^45^,^46^ for the sake of an extended comparison also within density functional theory itself. All DFT calculations are performed with the Q‐Chem program package.^47^

For the force field calculations we fall back on the GAFF^48^ and CGenFF v3.0.1^49^,^50^ force fields, which were specifically developed for the study of small drug molecules. The GAFF force field simulations are carried out with the LAMMPS program package,^51^ while CGenFF simulations are performed with the CHARMM program package.^52^ GAFF atom type assignments and the calculation of the partial charges are done with Antechamber^53^ package. For CGenFF, the atom type assignment, the derivation of partial charges, and other parameter settings are done with the CGenFF automation tool.^54^,^55^

We further test GFN‐xTB, a very recent semiempirical tight‐binding approach developed by the Grimme group for the particular purpose of noncovalent interactions in large molecular systems.^56^ According to its inventors, special focus during development has been put on a realistic description of structural features. Given the extremely sensitive dependence of barrier heights during propagation on the actual shape of the formed complex, this aspect is crucial to realistic predictions of separation tendencies in the given problem set.

In the calculations on complex formation, the aug‐cc‐pVDZ basis set^57^ is used for the geometry optimizations of all binary complexes at the DFT level of theory. For the force field calculations, a cutoff distance of 12 Å is chosen for electrostatic and van‐der‐Waals interactions, which is sufficiently large to include all atoms of any given molecular complex. For the studies on membrane propagation, we use the cc‐pVDZ basis set for all atoms of the pore and the cc‐pVQZ^58^ for the gas molecules. The DFT energies are corrected for basis set superposition errors (BSSE) using the method of Boys and Bernardi.^59^ Different basis set sizes are used for the pore and the target molecules in order to keep the BSSE as low as possible. With the current choice, it is never larger than 0.6 kcal/mol. In the pore scenarios, the force field cutoffs for GAFF are chosen sufficiently large to cover interactions between all atoms. For CGenFF, a cutoff of 12 Å has been employed since the force field was parametrized for this choice.

## 3 Tests on Complex Formation

**Figure 1:**
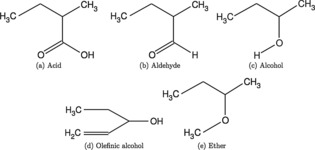
A selection of chiral organic molecules, tested for size differences when forming binary complexes from fragments of same or opposite chirality.

The separation mechanism proposed in Ref.[3] is based on the formation of a temporary complex between a free chiral molecule and a chiral ‘gatekeeper’, which is permanently attached to the rim of the pore. It could be shown that the ability to pass through such a pore is crucially depending on the size of this complex, since it has to swing through the pore in the case of a successful propagation event. An obvious consequence of this finding is that any separation performance will be determined by the size difference between complexes formed by the gatekeeper molecule and a left‐ or right‐handed drug molecule in solution. With this in mind, the purpose of this first study is two‐fold: On one hand, we aim to identify an appropriate low‐cost method for future studies on larger pore structures or subsequent MD simulations. On the other hand, we also investigate the interplay between sterical hindrance and the optimum alignment of attractively interacting functional groups of the temporary complex. Aiming for best separation results, we seek combinations with a maximum change of complex size between same‐chirality and opposite chirality pairings. Our test set consists of organic chiral template molecules as illustrated in Figure 1: Organic acid, aldehyde, alcohol, olefinic alcohol and ether templates are combined into bimolecular complexes. We distinguish between complexes formed by molecules of same (SS) and opposite chirality (RS).

**Figure 2:**
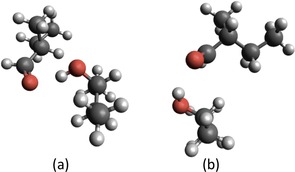
Different minimum geometries obtained for the RS complex (a) and the SS complex (b) formed by a chiral aldehyde and a chiral alcohol.

First, we identify the minimum energy configuration of each complex (see Figure 2) via geometry optimizations starting from several manually selected initial geometries based on chemical intuition. For each method the geometry of lowest energy is chosen. We compare the sizes and energies of the bimolecular complexes obtained with the various functionals, the tight‐binding approach and the two selected force fields. The size of a complex, denoted as *R*, is defined as the length between the two most distant atoms of the complex. Size differences ΔR and energy differences ΔE are calculated between the lowest minima of both enantiomeric combinations, i. e. according to the formulae

(1) $$\Delta E=E_{\mathrm{RS}}-E_{\mathrm{SS}},$$

(2) $$\Delta R=R_{\mathrm{RS}}-R_{\mathrm{SS}},$$

with R and S denoting the chirality of each monomer in the complex. Note that, for the proposed mechanism, large differences ΔR are particularly important, as they translate into large differences in the energy barriers which occur during the propagation of the mobile phase through the functionalized, porous membrane.

We start with the discussion of the results obtained with the three density functionals as presented in the first three columns of Tables 1 and 2. A clear correlation can be seen between the two van der Waals corrected functionals in energy as well as in size differences. B3LYP results deviate significantly from the other two functionals due to the lack of a long‐range correction. For most complexes, the energy differences ΔE are very small except for the group of alcohol‐acid complexes, followed by the group of olefinic alcohol‐acid combinations. An analysis of complex diameters reveals that the acid‐aldehyde, the acid‐alcohol and aldehyde‐aldehyde combinations show a large difference in size with respect to same‐chirality or opposite‐chirality pairings, which makes them particularly interesting for the proposed separation mechanism.

**Table 1:** Energy di*ff*erences *▵E* (in kcal*/*mol) between RS and SS bimolecular complexes according to Equation 1, obtained with various methods.

bimolecular complex	B3LYP	B97‐D	B97M‐V	GFN‐xTB	CGenFF	GAFF
alcohol‐alcohol	0.06	0.14	−0.21	−1.41	1.23	0.08
acid‐acid	0.32	0.28	0.36	0.01	0.00	1.68
aldehyde‐aldehyde	−0.17	0.03	−0.72	−0.48	0.12	−0.65
ether‐ether	2.02	1.00	1.67	0.09	2.22	1.17
olef. alcohol‐olef. alcohol	0.93	−0.75	0.78	1.08	−1.28	−1.93
alcohol‐acid	−6.58	−4.33	−5.70	−9.84	−5.44	−6.31
alcohol‐aldehyde	−0.51	−0.96	−0.12	−0.18	−1.91	0.79
alcohol‐ether	0.42	−0.16	0.31	−0.75	1.10	1.53
alcohol‐olef. alcohol	0.25	−0.92	−0.42	−1.32	−1.51	0.55
acid‐aldehyde	0.01	−0.17	−0.03	−0.23	0.29	0.05
acid‐ether	1.62	0.44	1.08	0.47	2.26	2.89
acid‐olef. alcohol	1.41	−1.92	−1.8	−1.53	−5.97	−3.49
aldehyde‐ether	0.14	0.27	0.68	0.32	0.54	0.72
aldehyde‐olef. alcohol	0.64	0.6	0.77	0.56	−0.13	0.13
ether‐olef. alcohol	−0.27	−0.22	−0.22	−0.17	−0.16	−0.52

**Table 2:** Size di*ff*erences *▵R* (in Å) between RS and SS bimolecular complexes according to Equation 2, obtained with various methods.

bimolecular complex	B3LYP	B97‐D	B97M‐V	GFN‐xTB	CGenFF	GAFF
alcohol‐alcohol	−0.29	0.04	0.06	0.19	−0.36	0.07
acid‐acid	−0.10	−0.05	−0.11	−0.04	−0.01	0.05
aldehyde‐aldehyde	0.18	0.79	0.54	0.39	0.29	1.71
ether‐ether	0.94	−0.41	−0.34	0.06	−0.28	−1.68
olef. alcohol‐olef. alcohol	−0.64	0.51	−1.04	−0.29	−0.37	1.30
alcohol‐acid	0.35	1.11	1.11	1.47	1.22	1.41
alcohol‐aldehyde	−0.35	0.35	−0.04	−0.67	1.57	0.14
alcohol‐ether	−0.62	0.40	0.42	−0.48	0.36	0.47
alcohol‐olef. alcohol	−1.21	−0.24	0.20	0.44	−2.40	0.24
acid‐aldehyde	0.35	−1.95	−1.67	−0.17	−0.08	−0.02
acid‐ether	−0.2	−0.39	−0.53	−1.22	0.34	0.38
acid‐olefinic alcohol	0.24	0.22	−0.28	0.54	0.04	−0.85
aldehyde‐ether	−0.21	−0.43	0.71	−0.05	−0.44	0.06
aldehyde‐olef. alcohol	0.56	−0.10	‐0.35	1.34	−0.03	1.04
ether‐olef. alcohol	−0.47	−0.54	−0.62	−0.35	−0.30	0.14

Results for the GFN‐xTB method and the two force fields, GAFF and CGenFF, are also listed in Tables 1 and 2. On first sight, the energy results for GAFF show almost no correlation to the DFT values. CGenFF seems to perform better, but the results still differ in many cases, e. g. for the acid ‐ olefinic alcohol complexes. However, this should not be overrated as most ΔE values lie within the energy variations observed within the group of DFT functionals. The size differences ΔR obtained with the GFN‐xTB approach and the two force fields do correlate for roughly one third of the complexes with the dispersion‐corrected DFT results. However, all three low‐cost alternatives clearly confirm the large size differences observed for the acid‐alcohol and aldehyde‐aldehyde combinations.

## 4 Tests on Membrane Propagation

**Figure 3:**
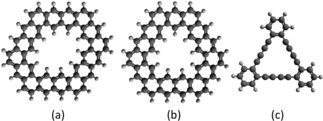
Pore models chosen for studies of molecule propagation: a) finite sheet of graphene with 4 benzene rings removed, b) finite sheet of graphene with 3 rings removed, and c) a model pore of graphdiyne.

Here we compare the chosen methods with respect to their predictions for approximative reaction pathways describing the propagation of methane, carbon dioxide, and ethane through three different pores illustrated in Figure 3. The free molecules, though not chiral, have been chosen as prototypes for an almost spherical electron density, an elongated, cigar‐shaped but radially symmetric density, and a corrugated electron density featuring also a slight constriction, respectively. For the porous membranes, represented by finite model pore environments due to computational limitations, we choose suitably sized pores derived from graphene or graphdiyne. The system size of chirally functionalized membranes, preferably with kinetic diameters in the range of the complexes discussed in Section 3, can be handled easily by force fields or the GFN‐xTB method, but is not accessible at the DFT level of theory. Therefore, in order to still provide a benchmark with the possibility for a direct comparison to DFT results, we refrain from a chiral functionalization in these preliminary tests. Instead, we choose pore sizes which provide insights into the general interplay between dispersion interaction and spatial hindrance due to Pauli repulsion. Although not directly representative for the proposed molecular sieving with respect to chirality, an agreement of much simpler reaction pathways is a minimum criterion for the actual applicability of a low‐cost method in future investigations of any type of pore propagation. The pores of our choice are slightly larger than the molecules chosen for our benchmark tests, with diameters between 5 and 6 Å.

**Figure 4:**
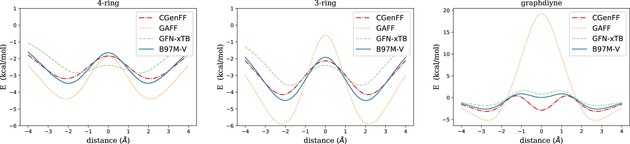
Propagation of carbon dioxide through various pores, evaluated with the B97M‐V functional and different force fields.

**Figure 5:**
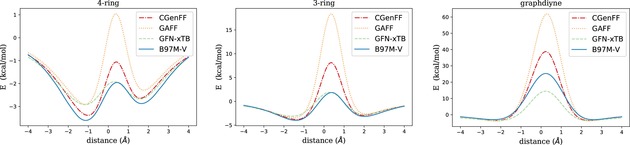
Propagation of methane through various pores, evaluated with the B97M−V functional and di*ff*erent force fields.

**Figure 6:**
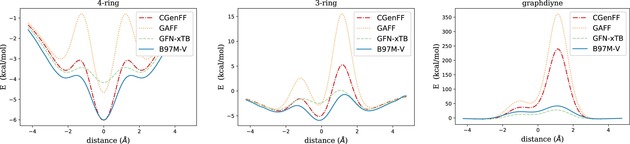
Propagation of ethane through various pores, evaluated with the B97M−V functional and di*ff*erent force fields.

In the case of the graphene‐based candidate pores, we include only those benzene rings which are necessary to define the pore shape. In the case of graphdiyne, we limit ourselves to a single triangular pore unit. Dangling bonds at the pore rim and the outer cutoff region are saturated with hydrogen atoms. Due to the larger pore diameters (6.0, 5.6 and 5.6 Å, respectively for 4‐ring, 3‐ring and graphdiyne) in comparison to the kinetic diameters of the target molecules,^60^ the reaction coordinate can be assumed to be close to the surface normal through the pore center. However, note that in this preliminary test we are less interested in the most accurate and realistic reaction pathway, but more on a direct comparison of energy predictions obtained with the different methods. Therefore, we will keep the pore geometries frozen at their global minimum structure obtained with the corresponding method, and push the molecules through the pore with their symmetry axis arranged perpendicular to the membrane surface. Also, no rotation of the molecules is allowed. This way, we will be able to judge the ability of the two force fields and the tight‐binding method to capture the most relevant features of this specific, one‐dimensional cut through the energy landscape as revealed at the DFT (B97M−V) level of theory. Obviously, the energy profiles obtained in such a constrained propagation are rather far from the minimum energy pathway, but they provide crucial information on effects such as Pauli repulsion (related to the shape of the molecular electron density) and sterical hindrance (related to the shape of the molecule itself). The corresponding scans of the total energy as a function of the distance between the center of mass of the target molecule and the pore center are presented in Figures 4, 5 and 6, for CO_2_, CH_4_ and C_2_ *H*_6_, respectively. The scans are performed with a step size of 0.25 Å per point. Both pore and target molecule are kept rigid during the scan. This measure enforces a well‐defined Cartesian reaction coordinate which allows a direct point‐wise comparison of the energy profiles obtained with different methods. The results are interpolated by cubic splines, and the point of zero energy is set to having pore and molecule infinitely separated. Note that all figures refer to electronic energies, i. e. no entropy and enthalpy corrections have been applied as we are concerned only with the ability of each method to reproduce the electronic potential surface obtained at the DFT level.

In the case of CO_2_, the energy profiles obtained with all methods are qualitatively the same for the 4‐ring and the 3‐ring pore. The energy profiles are fully symmetric due to the D${}_{\infty h}$ symmetry of the carbon dioxide molecule. With a kinetic diameter of 3.3 Å,^61^ the linear molecule moves through the two larger pores without a barrier in absolute energy, i. e. even the transition state has a negative energy (at least with zero point energy and thermochemistry corrections neglected). For all methods, adsorption minima occur outside of the pore center, with positions varying by less than 0.5 Å on average. While CGenFF and GFN‐xTB seem to slightly underestimate the oscillative character of the potential, GAFF is clearly overestimating potential depths and barrier heights during propagation. This becomes particularly evident for the smallest system, the graphdiyne pore, whith GAFF delivering an unphysically large energy barrier. CGenFF, on the other hand, shows a very good agreement with B97M−V and GFN‐xTB except for an overemphasized local minimum at z=0 , i. e. with the C atom exactly inside the pore.

For CH_4_ an asymmetric profile is obtained with all methods. The methane molecule, in Breck's popular book on molecular sieves listed with a kinetic diameter of 3.8 Å,^61^ moves through the pore from left to right with one of its C−H intermolecular axes perpendicular to the membrane. The tip of the tetrahedron passes first, giving rise to a global minimum on the left side of the pore (negative *z*) in all cases. With regards to potential energies, GAFF clearly overestimates the barriers, especially for the small graphdiyne pore. CGenFF also overestimates the barriers slightly, while GFN‐xTB is showing a reasonable agreement with the DFT pathway for the two larger pores (apart from a slight underestimation of the minima). For graphdiyne, GFN‐xTB is underestimating the barrier, but to a lesser extent as CGenFF is overestimating it.

In the case of C_2_ *H*_6_, the profile is slightly more complicated due to the stepwise interactions of the two methyl groups with the pore rim. Surprisingly, CGenFF is getting closest to the B97M‐V reference profile for the two larger pores, even outperforming GFN‐xTB, which shows a tendency to underestimate local minima of intermediate states. However, the conceptual breakdown of both force field methods in the case of graphdiyne makes again GFN‐xTB the method of choice. One might argue that the unphysical results for barriers obtained with force fields are artificial and ‘unfair’ in the sense that we enforced a rigid pore and molecule structure during propagation, but our focus is set on a physically correct description of these extreme cases of sterical hindrance for the sake of trustworthy barriers in the following studies of membrane‐based chiral resolution.

## 5 The Separation Principle Revisited

We revisit the original system which had been chosen for the initial demonstration of the separation principle based on a gatekeeper molecule.^3^ A finite pore model consisting of 17 benzene rings had been functionalized with a permanently attached 1‐aminoethanol molecule to the pore rim. The pore itself is formally obtained by the removal of 5 rings from pristine graphene and a subsequent passivation of dangling bonds with hydrogen atoms.

**Figure 7:**
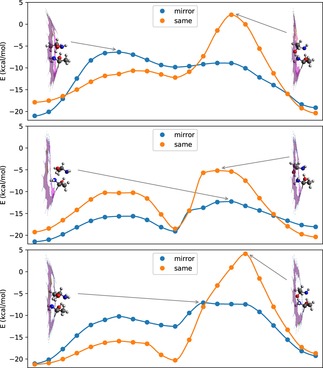
Reaction pathways for the propagation of 1‐aminoethanol through a chirally functionalized pore: a) original pore from Ref. 3, b) same pore with two C atoms opposite of the gatekeeper replaced by N, c) same pore with C atoms next to the gatekeeper replaced by N atoms.

In a first step, we calculate the reaction pathway for the propagation of a free 1‐aminoethanol molecule without any further constraints at the GFN‐xTB level of theory via the Nudged Elastic Band Method as implemented in the ASE suite of programs.^62^ In Table 3, the outcome is directly compared to the DFT electronic energy results as presented in Ref. [3]. As can be seen, the tight‐binding approach is able to deliver a reaction pathway in excellent agreement with the DFT results, but with a fraction of the original computational cost. It confirms the proposed chiral selectivity, but also the relatively high barrier for propagation of the enantiomer forming the smaller temporary complex. These qualitative features of the reaction pathway stay the same also after the inclusion of thermochemistry corrections; see Ref. [3] for the corresponding Gibbs energy pathways. Therefore, in a next step, we introduce minimal modifications to the original pore size. A convenient way of pore adjustment is the replacement of a carbon atom and its corresponding H atom at the rim by a nitrogen atom,^63^,^64^ which leads to a minimal enlargement of the pore area. We choose two conceptually different replacement scenarios (see small graphics inside of Figure 7): In one case, we replace two C atoms opposite of the gatekeeper molecule, which simply enlarges the pore area. In the other case, we replace two C atoms next to the gatekeeper, which has an almost negligible influence of the pore size due to the presence of the gatekeeper on this side of the pore, but gives the gatekeeper more flexibility with respect to the swinging motion necessary for the propagation of the target molecule. As can be seen in Figure 7, which also contains the corresponding reaction pathways as obtained in all three cases, both replacement strategies have a significant impact on the barrier height of the two transition states which occur during propagation. Interestingly, the modification with N atoms next to the gatekeeper (lowest graph of Figure 7) is mostly affecting the first transition state, which seems less related to the actual propagation of the target molecule itself but more with a rearrangement of the gatekeeper. The second transition state, which is now the rate determining step for both enantiomers, is almost of the same height as in the unmodified case, independent on the actual chirality of the molecule. It follows that, by comparison of absolute as well as relative barrier heights, this modification is ineffective in terms of permeance as well as chiral selectivity. However, the second modification, where C atoms opposite of the gatekeeper have been replaced, affects both transition states (see middle graph of Figure 7) and seems to be of greater relevance for a fine‐tuning of the propagation with respect to the chirality of the target molecule. We observe a comparable difference of relative energies between the rate determining steps for the two enantiomers, which indicates a comparable selectivity, but significantly lower absolute barrier heights in the case of the nitrogen‐functionalization. This suggests a much higher permeance, and makes this pore structure an even better candidate for membrane‐based chiral resolution. However, we note that our predictions are based only on the evaluation of one‐dimensional reaction pathways on the electronic PES. Future studies on more realistic systems will have to account for dynamic effects such as inter‐particle interactions, changing adsorption probabilities and entropic barriers,^65^,^66^ preferably by combining molecular dynamics simulations with stochastic transition state theory models.^67^

**Table 3:** Comparison of extrema on the reaction pathway for the propagation of 1‐aminoethanol through a chirally functionalized pore, calculated with B97M−V and GFN‐xTB.

	opp. chirality		same chirality	
geometry	DFT	xTB	DFT	xTB
left minimum	−21.37	−21.00	−20.26	−17.88
1${}^{\mathrm{st}}$ transition state	−8.34	−6.40	−8.99	−10.66
intermediate	−10.95	−9.83	−9.44	−12.21
2${}^{\mathrm{nd}}$ transition state	−10.43	−8.89	−0.27	2.16
right minimum	−18.00	−19.12	−21.14	−20.36

## 6 Conclusions

Chiral resolution based on single‐atom thick porous membranes is a novel concept of molecular sieving, which takes advantage of small differences in the size of a temporary complex formed by the chiral target molecule and a chiral functionalization which is permanently attached to the rim of the pore. The computational study of this sieving technique is challenging due the large system size in combination with the necessity to describe spatial hindrance at a resolution which is highly problematic for most force fields. In a first step, we analyzed which combinations of chiral template molecules in organic chemistry lead to largest differences in the size of the complexes formed by two enantiomers of same or of opposite chirality. We compared the outcome of various density functionals to the results obtained the GFN‐xTB method and the two force fields GAFF and CGenFF. Although an agreement of density functionals with dispersion corrections among themselves is obvious and also partially recovered by the less costly methods, the geometry of the complexes varies still so strongly with the method chosen that we refrain from an actual ranking of suitability of template structures. However, particularly large size differences for the acid‐alcohol and aldehyde‐aldehyde bimolecular complexes are confirmed by all methods applied in this study. Therefore, we recommend to focus on these combinations of template structures for future experimental investigations of the proposed separation mechanism.

We further tested the two force fields and the GFN‐xTB approach for their general ability to reproduce approximate reaction pathways for molecules propagating through porous membranes. Pore and molecule geometries were kept frozen at their minimum energy structures for the sake of a direct comparison of the interplay between attractive dispersion interaction and Pauli repulsion as a function of the distance between pore and free molecule. As expected, force field‐based approaches tend to overestimate transition barriers related to spatial hindrance. Especially in cases where the pore radii become comparable to the kinetic diameters of the free molecules large deviations in the range of an order of magnitude become apparent. This unphysical behavior at non‐equilibrium geometries makes the tested force field approaches less suitable for realistic descriptions of the proposed separation mechanism. Fortunately, this problem can be circumvented by the only slightly more costly GFN‐xTB method, which will be the method of choice for our future investigations of chiral resolution on two‐dimensional membranes. However, we note that the performance of the CGenFF, apart from its systematic error in the cases discussed above, is actually comparable to that of GFN‐xTB and in several cases even closer to the DFT energy profiles.

An obvious next step is to use the tight‐binding approach for molecular dynamics simulations of porous two‐dimensional materials for chiral resolution. From these future studies we expect more realistic estimates for selectivities as well as for the particle flux, as they will sample over large parts of the relevant phase space instead of relying on a single, idealized trajectory. Also, membrane adsorption, pore blocking and solvent effects need to be taken into consideration in follow‐up studies.

## Conflict of interest

The authors declare no conflict of interest.
